# Enhanced Production of a Thermostable Carbonic Anhydrase in *Escherichia coli* by Using a Modified NEXT Tag

**DOI:** 10.3390/molecules26195830

**Published:** 2021-09-26

**Authors:** In Seong Hwang, Joo Hyeon Kim, Byung Hoon Jo

**Affiliations:** 1 Division of Applied Life Science, Gyeongsang National University, Jinju 52828, Korea; inseong25@naver.com; 2 Division of Life Science, Gyeongsang National University, Jinju 52828, Korea; rlawngus4858@naver.com; 3 Division of Life Science and Research Institute of Life Science, Gyeongsang National University, Jinju 52828, Korea

**Keywords:** recombinant protein, solubility enhancer, NEXT tag, carbonic anhydrase, *Thermovibrio ammonificans*, CO_2_ capture

## Abstract

Carbonic anhydrase (CA) is an ultrafast enzyme that catalyzes the reversible conversion of carbon dioxide (CO_2_) to bicarbonate. CA is considered to be a green catalyst for enzyme-based CO_2_ capture and utilization. In particular, the CA of *Thermovibrio ammonificans* (taCA) has attracted increasing attention as a highly stable enzyme. However, the poor solubility and the low expression level in *Escherichia coli* have hampered further utilization of taCA. In a recent study, these limitations were partly resolved by using a small solubility-enhancing fusion tag named NEXT, which originates from the N-terminal extension of *Hydrogenovibrio marinus* CA. In this study, the NEXT tag was engineered by adding small peptides to the N terminus to further increase the production yield of NEXT-tagged taCA. The addition of ng3 peptide (His-Gly-Asn) originating from the N-terminal sequence of *Neisseria gonorrhoeae* CA improved the expression of NEXT-taCA, while the previously developed translation-enhancing element (TEE) and Ser-Lys-Ile-Lys (SKIK) tag were not effective. The expression test with all 16 codon combinations for the ng3 sequence revealed that the change in translation initiation rate brought about by the change in nucleotide sequence was not the primary determinant for the change in expression level. The modified ng3-NEXT tag may be applied to increase the production yields of various recombinant proteins.

## 1. Introduction

Carbonic anhydrase (CA, EC 4.2.1.1) is a ubiquitous (metallo) enzyme that plays important roles in various physiological processes, such as carbon dioxide (CO_2_) transport, CO_2_ metabolism, and pH homeostasis, by catalyzing the reversible hydration of CO_2_: CO_2_ + H_2_O ↔ HCO_3_^−^ + H^+^ [1]. CAs are classified into eight families, including the recently discovered ι-CA family, that are evolutionarily distinct [2]. CAs are diffusion-limited enzymes with a *k*_cat_ of up to 4.4 × 10^6^ s^−1^ [3]. Due to the ultrafast kinetics, CA is considered to be a powerful biocatalyst for CO_2_ capture and utilization (CCU) technologies to respond to climate change [4]. By accelerating CO_2_/HCO_3_^−^ interconversion, CA can supply a sufficient amount of the inorganic carbon species as a feedstock for formic acid or methanol production [5,6], in vitro carboxylation [7], microalgal cultivation [8], and mineral carbonation [9]. For industrial utilization of CA, however, the low stability of CA and the high cost of CA production should be overcome.

The CA of *Thermovibrio ammonificans* (taCA), which originated from a deep-sea hydrothermal vent, has been expressed and purified in an *Escherichia coli* host [10,11,12]. A study on the purified recombinant taCA has demonstrated that taCA is the most thermostable bacterial CA with a half-life of 77 days at 60 °C under an aqueous buffer condition, making this stable enzyme a promising candidate for enzyme-based CCU [10]. However, the production yield of taCA in *E. coli* was relatively low, and, more seriously, the purified taCA showed poor solubility under low salt conditions, resulting in protein aggregation and precipitation [10].

Solubility-enhancing proteins can be used as fusion tags not only for circumventing the poor solubility but also for improving the expression level of target proteins [13]. Recently, a small but powerful solubility enhancer, named the NEXT tag, was developed [14]. The NEXT tag is a protein that is 53 amino acids in length, has a molecular mass of 5.5 kDa, and originated from the N-terminal extension of *Hydrogenovibrio marinus* CA. The NEXT tag is an intrinsically disordered protein that can entropically exclude large particles around its point of attachment to a target protein by random movements, preventing protein aggregation [14,15,16]. Despite its small size, the ability of the NEXT tag to improve both the solubility and production yield of target proteins was found to be superior to that of conventional tags, such as maltose-binding protein (MBP) with a molecular mass of 40.4 kDa. By the N-terminal fusion of the NEXT tag, a high-level expression of soluble taCA was achieved and no precipitation of purified enzyme was observed. In addition, the NEXT tag only minimally affected the activity and stability of taCA, while the inherent properties of taCA were abnormally altered by fusion to the large MBP. The outstanding ability of the NEXT tag prompted us to engineer the NEXT tag for more efficient production of target proteins.

Protein engineering of the NEXT tag by directed evolution can be highly effective at finding improved variants; however, it is generally time-consuming and labor-intensive. The design and introduction of a point mutation for improving protein expression is virtually impossible due to our poor understanding of the influence of such a mutation on the overall expression level of the target protein. In addition, these approaches can add another level of complexity since any amino acid substitution can potentially affect the function of the NEXT tag. Because protein expression is controlled primarily in the N-terminal coding region as well as in the 5′ untranslated region [17], a change in amino acid sequence at the N terminus of the protein, as exemplified by the N-terminal fusion of small peptide tags, can drastically affect the expression level [18,19,20]. Based on this simple idea, herein, the N-terminal fusion of short peptides is examined to engineer the NEXT tag and improve the expression level of NEXT-tagged proteins, particularly NEXT-taCA. We show that the tripeptide His-Gly-Asn derived from the N-terminal sequence of a highly expressed CA of *Neisseria gonorrhoeae* is effective at improving the expression level of NEXT-tagged target proteins.

## 2. Materials and Methods

### 2.1. E. coli Strains and Plasmid Vector Construction

The strains, plasmids, and oligonucleotide primers used in this study are listed in Table 1. *E. coli* TOP10 (Thermo Fisher Scientific, Waltham, MA, USA) was used for the construction of plasmid vectors, and *E. coli* BL21(DE3) (Merck Millipore, Burlington, MA, USA) was used for recombinant protein expression. The cells were routinely cultivated in Luria-Bertani (LB) medium supplemented with appropriate antibiotics (50 μg/mL of ampicillin for recombinant strains and 10 μg/mL of streptomycin for wild-type *E. coli* TOP10) at 37 °C and 180 rpm in a shaking incubator (Jeiotech, Daejeon, Korea). The genes for modified NEXT tags were amplified by polymerase chain reaction (PCR) (Takara Bio, Shiga, Japan) using the primers listed in Table 1 and the previously constructed pET-NEXT-taCA [14] as the template. The PCR products were ligated into the pGEM-T Easy vector (Promega, Madison, WI, USA) and the insert sequences were confirmed by direct sequencing. The genes were subcloned into pET-NEXT-taCA and pET-NEXT-GFP treated with *Nde*I and *Nco*I restriction enzymes, substituting the original NEXT tag sequence with the modified ones. All of the recombinant proteins have a hexahistidine (His_6_) tag at the C terminus and they were expressed under the control of T7*lac* promoter. The taCA gene was codon-optimized for *E. coli* with a codon adaptation index (CAI) of 0.88 [10]. The DNA and protein sequences of NEXT-taCA are shown in Table 2.

### 2.2. Recombinant Protein Expression

Recombinant *E. coli* BL21(DE3) strains transformed with the constructed plasmids were incubated in Luria-Bertani medium supplemented with 50 μg/mL of ampicillin at 37 °C in the shaking incubator. The expression of recombinant protein was induced at 0.6–0.8 OD_600_ by adding 1 mM isopropyl-β-D-thiogalactopyranoside (Duchefa Biochemie, Haarlem, The Netherlands). In addition, a sufficient amount of zinc was supplemented as the enzyme cofactor by adding 0.1 mM ZnSO_4_ (Junsei, Tokyo, Japan). The cells were further cultivated at 37 °C for 12 h, followed by centrifugation at 4 °C and 4000× *g* for 10 min and resuspension in lysis buffer (50 mM sodium phosphate, 300 mM NaCl, and 10 mM imidazole; pH 8.0). The cell lysate was prepared by disrupting the cell suspension with an ultrasonic dismembrator (Sonics and Materials, Newtown, CT, USA) for 10 min at 20% amplitude (a 2-s pulse on and a 10-s pulse off) in ice water. The lysate was centrifuged at 4 °C and 10,000× *g* for 10 min, and the supernatant was designated the soluble fraction (S), while the pellet was designated the insoluble fraction (IS).

### 2.3. Recombinant Protein Purification

Recombinant protein was purified by immobilized metal affinity chromatography (IMAC) via the His_6_ tag. After cell lysis, the soluble fraction was mixed with Ni^2+^-nitrilotriacetic acid agarose beads (Qiagen, Hilden, Germany), and the recombinant protein was purified according to the manufacturer’s instructions. The purified protein was thoroughly dialyzed against 20 mM sodium phosphate buffer supplemented with 300 mM NaCl (pH 7.5) at 4 °C.

### 2.4. Protein Gel Electrophoresis and Western Blot

Samples were separated by sodium dodecyl sulfate-polyacrylamide gel electrophoresis (SDS-PAGE) on 15% PAGE gel and visualized by Coomassie blue R-250 (Bio-Rad, Hercules, CA, USA) staining. For Western blot, the separated samples were blotted onto a nitrocellulose membrane (Whatman, Maidstone, UK). Monoclonal anti-His_6_ antibody (ABM, Richmond, BC, Canada) and alkaline phosphatase-conjugated anti-mouse IgG (Bethyl Laboratories, Montgomery, TX, USA) were sequentially treated. Target proteins were visualized using nitroblue tetrazolium and 5-bromo-4-chloro-3-indolyl phosphate (NBT/BCIP; Sigma-Aldrich, St. Louis, MO, USA).

### 2.5. CO_2_ Hydration Assay

CA activity was measured by CO_2_ hydration assay based on the time-course pH change estimated by using phenol red (Sigma-Aldrich, St. Louis, MO, USA) as a pH indicator [21]. An appropriate amount of diluted sample (usually 10 μL) was mixed with 600 μL of 20 mM Tris buffer (pH 8.3) supplemented with 100 μM phenol red in a disposable cuvette. Four hundred microliters of ice-cold CO_2_-saturated deionized water was added and mixed by thorough pipetting. The reaction was performed at 4 °C inside a UV-Visible spectrophotometer (Shimadzu, Kyoto, Japan) and the time-course absorbance change was monitored at 570 nm. The noncatalyzed reaction was measured by adding the same amount of blank buffer instead of enzyme solution. The time (*t*) required for the absorbance to decrease from 1.2 (corresponding to pH 7.5) to 0.18 (corresponding to pH 6.5) was obtained. The Wilbur–Anderson unit (WAU) was calculated as (*t*_0_ − *t*)/(*t* × 5), where *t*_0_ is the time for the noncatalyzed reaction [21,22].

### 2.6. Quantification of Purified Protein

The purified enzyme was denatured in denaturing buffer (6 M guanidine hydrochloride/20 mM sodium phosphate buffer; pH 7.5), and the absorbance of the denatured protein at 280 nm was measured in a quartz crystal cuvette (Hellma Analytics, Müllheim, Germany). The protein concentration of the purified sample was determined using the measured absorbance and the calculated extinction coefficient at 280 nm by ProtParam (http://web.expasy.org/protparam/) [23].

### 2.7. Thermostability Test

The concentration of purified enzyme was adjusted to 10 μM. The enzyme was incubated at 80 °C for 12 h and then immediately cooled on ice. The CO_2_ hydration activity of the incubated sample was measured and compared with the activity of the nonincubated sample. The relative activity of the incubated sample was calculated and is presented as residual activity (%).

### 2.8. Fluorescence Measurement

The lysates of green fluorescent protein (GFP)-expressing cells were used for the measurement. The GFP fluorescence was measured with excitation and emission wavelengths at 430 nm and 510 nm, respectively, using a microplate reader (Tecan, Männedorf, Switzerland).

### 2.9. In Silico Analyses

Densitometric analysis of the protein band was performed using ImageJ [24]. Prediction of expression level was done by the UTR designer (https://sbi.postech.ac.kr/utr_designer/) [25].

## 3. Results and Discussion

### 3.1. Effects of N-Terminal Addition of Translation-Enhancing Element and SKIK Tag

**Figure 1:**
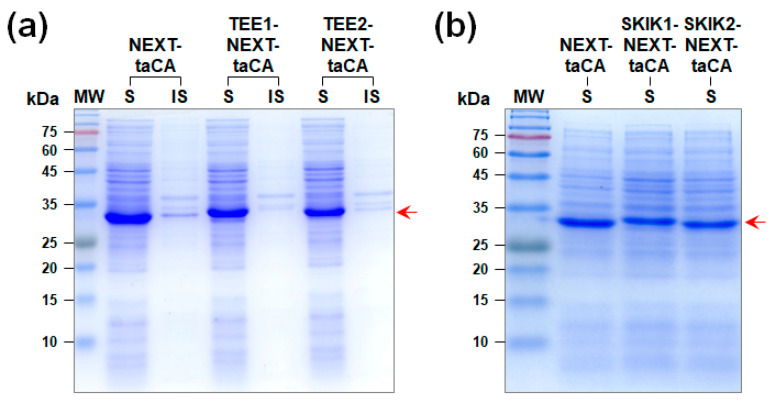
Expression of NEXT-taCA fused with (**a**) the translation-enhancing element (TEE) or (**b**) the SKIK tag analyzed by SDS-PAGE followed by Coomassie blue staining. The arrow indicates the band positions of recombinant proteins. Lanes: MW, molecular mass marker; S, soluble fraction; IS, insoluble fraction.

The translation-enhancing element (TEE), also known as the downstream box, is a cis element downstream of the start codon that can enhance the translation initiation by complementarily binding to a region of 16S rRNA [26]. The TEE sequence consists of 5′-AATCACAAAGTG-3′, which corresponds to the amino acid sequence of Asn-His-Lys-Val. As the first trial, the TEE sequence was added to the N terminus of NEXT-taCA right after the start codon. We tested both the direct fusion of the TEE sequence to the NEXT tag (TEE1) and the replacement of the first four amino acids of the NEXT tag with the TEE sequence (TEE2). Unfortunately, the expression level of NEXT-taCA (~33.1 kDa) decreased by 31% (TEE1) and 43% (TEE2) after the fusion of the TEE sequence (Figure 1a).

The Ser-Lys-Ile-Lys (SKIK) tag is a short peptide that was developed based on the most frequently found amino acid at each position in the four N-terminal amino acid positions excluding the first Met in highly expressed *E. coli* proteins [20]. Similar to the case of TEE fusion, we examined the effect of addition of the SKIK tag to the N terminus of NEXT-taCA by direct fusion (SKIK1) or by replacement of the N-terminal sequence of the NEXT tag (SKIK2). Again, both the constructs with the SKIK tag resulted in the decreased production of NEXT-taCA (Figure 1b). Although the TEE and SKIK sequences have been successfully used for improving the production of other recombinant proteins, they appeared to negatively affect the expression of NEXT-tagged proteins when combined with the NEXT tag, presumably by reducing the efficiency of the NEXT tag for gene expression.

### 3.2. Improved Production of NEXT-taCA by Fusion with a Peptide Derived from ngCA

The CA from *N. gonorrhoeae* (ngCA) is one of the most extensively studied bacterial CAs [27,28]. The ngCA is a highly soluble protein and its high-level expression in *E. coli* has been successfully demonstrated in previous studies [29,30]. Inspired by the high-level expression of soluble ngCA, the N-terminal sequences of ngCA were examined as fusion tags for improving the expression of NEXT-taCA.

**Figure 2:**
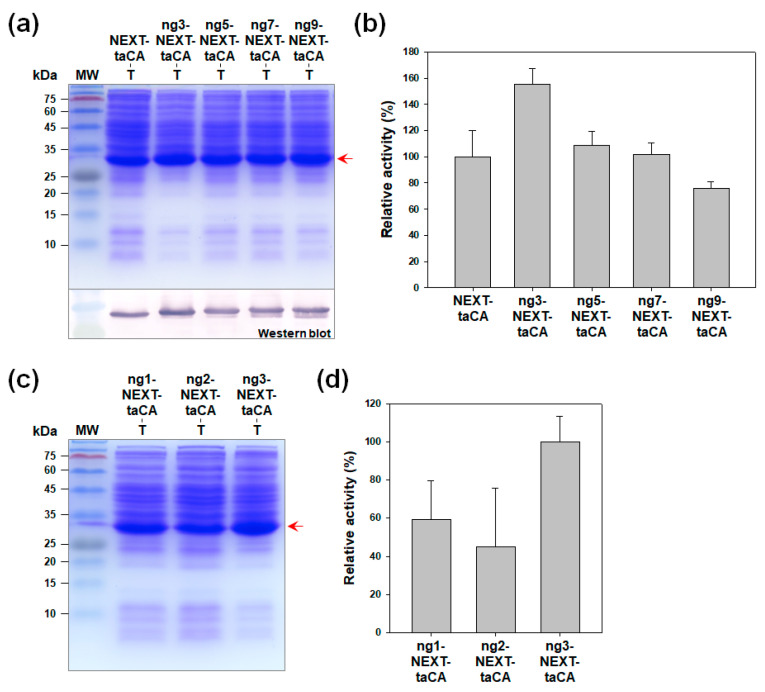
Effect of fusion of ngCA-derived peptide tags to NEXT-taCA. (**a**,**c**) Expression analysis of modified NEXT-tag-fused taCA by SDS-PAGE followed by Coomassie blue staining. The arrow indicates the band position of recombinant proteins. Western blot was performed using an anti-His_6_ antibody. Lanes: MW, molecular mass marker; T, total cell lysate; (**b**,**d**) Relative CO_2_ hydration activities of whole-cell lysates. Error bars represent standard deviations from two independent experiments.

At first, the peptides that consist of three (ng3), five (ng5), seven (ng7), and nine (ng9) N-terminal amino acids of ngCA excluding the first Met were tested as fusion tags. The nine amino acids with a sequence of His-Gly-Asn-His-Thr-His-Trp-Gly-Tyr (HGNHTHWGY) correspond to 27 nucleotides, which is long enough to cover the coding region in the ribosome docking site and thus affect the translation initiation rate [17]. The expression of the four variants along with the unmodified NEXT-taCA revealed that the ng3-NEXT-taCA, fused with the HGN sequence, showed the highest expression level and was ~36% more produced than the unmodified control as analyzed by densitometric quantification (Figure 2a). The catalytic activities for CO_2_ hydration were measured using the cell lysates, and the highest activity, which was ~55% higher than that of the unmodified control, was obtained when using the lysate of ng3-NEXT-taCA (Figure 2b). We further tested whether one amino acid (ng1, His) or two amino acids (ng2, His-Gly) of ngCA would be better than the ng3 sequence. The expression levels and the activities of cell lysates of both ng1- and ng2-fused NEXT-taCA were reduced compared with those of the ng3-NEXT-taCA (Figure 2c,d). These results demonstrate that the addition of the HGN sequence to the N terminus was optimally effective for improving the production yield of NEXT-taCA.

**Figure 3:**
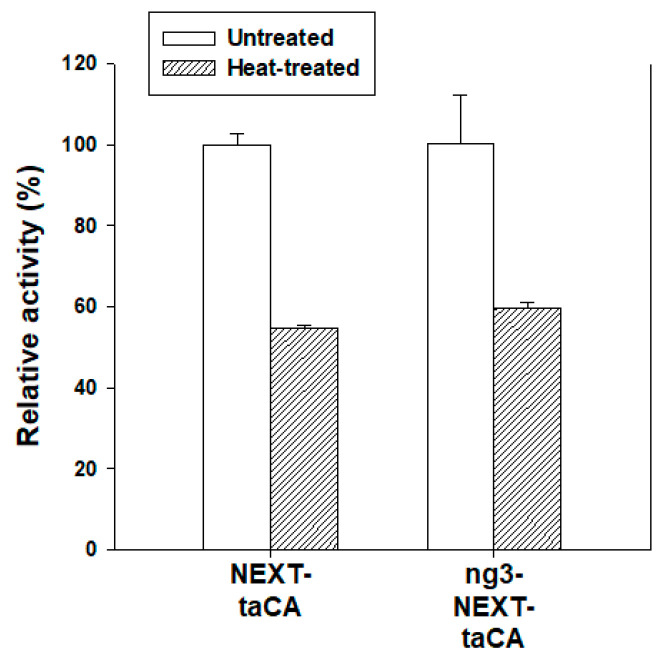
Characterization of purified enzymes. Activities (%) of purified enzymes normalized by the activity of untreated NEXT-taCA are presented before and after heat treatment at 80 °C for 12 h. Error bars represent standard deviations from two independent experiments.

The catalytic activity of a cell lysate can be increased not only by an increased production of enzyme but also by an increase in the specific activity of the enzyme. Because it would be possible that the addition of HGN alters the structure and, in turn, the specific activity of NEXT-taCA, we purified the ng3-NEXT-taCA along with the original NEXT-taCA from the lysate by IMAC and measured the specific activity of the enzyme for CO_2_ hydration. The specific activity of NEXT-taCA was not altered by the fusion of the HGN sequence (Figure 3), implying that the increased enzymatic activity of the cell lysate was exclusively due to the improved enzyme production. In addition, the thermal stability of NEXT-taCA also was not significantly affected by the addition of the HGN sequence (Figure 3). Thus, it can be concluded that the modified NEXT tag with the additional HGN sequence successfully improved the expression level compared with the unmodified control with a minimal influence on the properties of the target protein.

**Figure 4:**
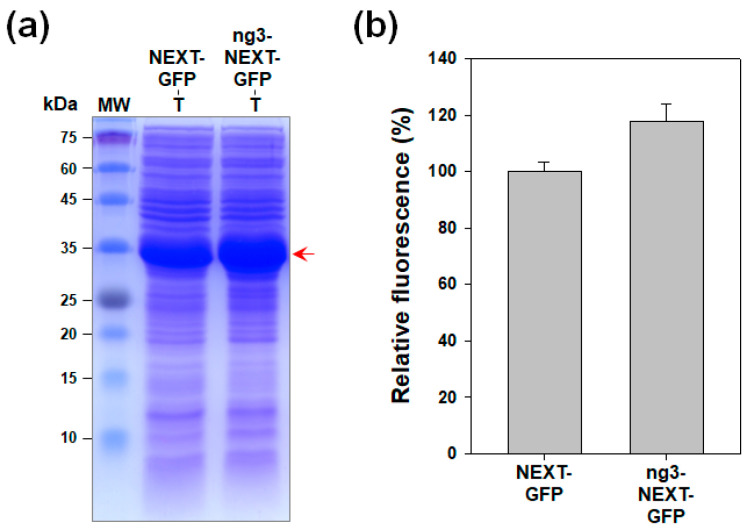
Effect of fusion of the ng3 sequence on the expression level of NEXT-GFP. (**a**) Expression analysis by SDS-PAGE followed by Coomassie blue staining. The arrow indicates the band position of recombinant proteins. Lanes: MW, molecular mass marker; T, total cell lysate. (**b**) Relative GFP fluorescence of whole-cell lysates. Error bars represent standard deviations from triplicate experiments.

To further test the applicability of the ng3-NEXT tag, we constructed ng3-NEXT-GFP and compared it with NEXT-GFP. The expression level of NEXT-GFP was successfully improved by the addition of ng3 peptide (Figure 4a). The GFP fluorescence intensity of the cell lysate of ng3-NEXT-GFP was also higher (18%) compared with that of NEXT-GFP (Figure 4b). This result suggests that the modified NEXT tag with the N-terminal ng3 sequence can be potentially used as a general tag for better expression of the target protein compared with the original NEXT tag.

### 3.3. Combinatorial Test of the Coding Sequence for the ng3 Peptide

The coding sequence for the ng3 peptide used in our experiment was 5′-CACGGCAAT-3′. Although the fusion of ng3 improved the production of NEXT-taCA, it was not clear whether the improved production was achieved in the context of the amino acid or the nucleotide sequence. To investigate the effect of using synonymous codons on the protein expression level and optimize the codon combination of the ng3 peptide, we designed all of the 16 different possible combinations of codons for the HGN sequence (c1 to c15), including the original one (c0), and constructed the corresponding vectors for the expression of ng3-NEXT-taCA (Table 1).

**Figure 5:**
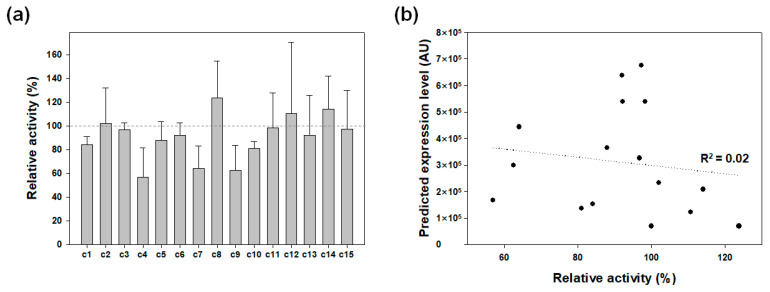
Effect of the codon combination of the ng3 sequence. (**a**) Relative activities of whole-cell lysates of ng3-NEXT-taCA-expressing cells with different codon combinations for the ng3 sequence. The activity of the lysate with the original codons (c0) for ng3 corresponds to the 100% relative activity, which is indicated with the dashed line. Error bars represent standard deviations from two or three independent experiments. (**b**) Relation between activities of cell lysates and predicted expression levels. The dashed line indicates linear regression. AU, arbitrary unit.

Because all of the variants with different codon combinations would preserve the encoded amino acid sequence and it is not likely that the synonymous codon substitutions would perturb the enzyme folding and structure, which in turn would alter the specific activity of the enzyme [31], it could be assumed that the CO_2_ hydration activity of the cell lysate would be proportional to the expression level of ng3-NEXT-taCA. The expression levels of the variants did not significantly deviate from that of the original ng3-NEXT-taCA, although some of them showed lower expression levels (Figure 5a). In addition, none of the codon combinations of ng3 clearly resulted in an improved expression of the enzyme compared with the original c0 (Figure 5a). Since it is generally accepted that protein expression is proportional to the rate of translation initiation [17], the translation initiation rates were predicted for the variants by using the UTR designer to compare them with the experimentally obtained expression levels (Table 3) [25]. Notably, the predicted rate for the original ng3-NEXT-taCA (70,038) was remarkably lower than that for NEXT-taCA (997,966) (Table 3), while ng3-NEXT-taCA showed a higher expression level compared with NEXT-taCA as previously shown (Figure 2). In addition, the analysis of the relation between the predicted rates of ng3-NEXT-taCA variants and the experimental results revealed that the two factors showed no linear correlation (Figure 5b). These results show that the change in translation initiation rate brought about by the change in codon combination (i.e., nucleotide sequence) was not the primary determinant for the change in expression level. The improved expression of NEXT-taCA by the fusion of ng3 was likely to be achieved in the context of the amino acid sequence, which is similar to the case of the SKIK tag where two completely different codon combinations for the SKIK tag showed no significant difference in the expression level of the target protein [20].

## 4. Conclusions

The fusion of tripeptide ng3 (HGN) to the N terminus of the NEXT tag was effective at improving the expression level without affecting the inherent properties of NEXT-taCA, while the previously developed sequences, such as TEE and SKIK, were ineffective. The amino acid sequence of ng3, and not the specific codon combination that would affect the translation initiation rate, appeared to be a primary determinant of the positive effect of ng3 addition on the protein expression level. In addition to the thermostable taCA that can be used for CO_2_ capture and utilization, we expect that the production yields of other industrially important proteins might be improved by using the ng3-NEXT tag in *E. coli*.

## Figures and Tables

**Table 1:** Strains, plasmids, and oligonucleotide primers used in this study.

Strains, Plasmids, and Primers	Genotypes, Relevant Characteristics and Sequences	Source or References
**Strains**		
*E. coli* TOP10	F- *mcrA* Δ(*mrr-hsdRMS-mcrBC*) Φ80*lacZ*ΔM15 Δ*lacX*74 *recA*1 *araD*139 Δ(*ara-leu*)7697 *galU galK rpsL*(Str^r^) *endA*1 *nupG*	Thermo Fisher Scientific
*E. coli* BL21(DE3)	F- *ompT hsdS*_B_(r_B_^−^ m_B_^−^) *gal dcm lon* λ(DE3), carrying the T7 RNA polymerase gene	Novagen
**Plasmids**		
pGEM-T Easy	pUC *ori*, Amp^r^, TA cloning vector	Promega
pET-22b(+)	T7*lac* promoter, pBR322 *ori*, Amp^r^, parental expression vector harboring the PelB signal sequence	Novagen
pET-NEXT-taCA	Expression plasmid carrying the NEXT-taCA fusion gene	[14]
pET-NEXT-GFP	Expression plasmid carrying the NEXT-GFP fusion gene	[14]
pET-TEE1-NEXT-taCA	Expression plasmid carrying the NEXT-taCA gene directly fused with the translation-enhancing element (TEE) sequence	This study
pET-TEE2-NEXT-taCA	Expression plasmid carrying the NEXT-taCA gene where the sequence for the four N-terminal amino acids is replaced with the translation-enhancing element (TEE) sequence	This study
pET-SKIK1-NEXT-taCA	Expression plasmid carrying the NEXT-taCA gene directly fused with the SKIK sequence	This study
pET-SKIK2-NEXT-taCA	Expression plasmid carrying the NEXT-taCA gene where the sequence for the four N-terminal amino acids is replaced with the SKIK sequence	This study
pET-ng1-NEXT-taCA	Expression plasmid carrying the NEXT-taCA gene fused with the H sequence	This study
pET-ng2-NEXT-taCA	Expression plasmid carrying the NEXT-taCA gene fused with the HG sequence	This study
pET-ng3-NEXT-taCA	Expression plasmid carrying the NEXT-taCA gene fused with the HGN sequence	This study
pET-ng5-NEXT-taCA	Expression plasmid carrying the NEXT-taCA gene fused with the HGNHT sequence	This study
pET-ng7-NEXT-taCA	Expression plasmid carrying the NEXT-taCA gene fused with the HGNHTHW sequence	This study
pET-ng9-NEXT-taCA	Expression plasmid carrying the NEXT-taCA gene fused with the HGNHTHWGY sequence	This study
pET-ng3(c1)-NEXT-taCA	Expression plasmid carrying the NEXT-taCA gene fused with the HGN sequence encoded by 5′-CACGGCAAC-3′	This study
pET-ng3(c2)-NEXT-taCA	Expression plasmid carrying the NEXT-taCA gene fused with the HGN sequence encoded by 5′-CACGGGAAT-3′	This study
pET-ng3(c3)-NEXT-taCA	Expression plasmid carrying the NEXT-taCA gene fused with the HGN sequence encoded by 5′-CACGGGAAC-3′	This study
pET-ng3(c4)-NEXT-taCA	Expression plasmid carrying the NEXT-taCA gene fused with the HGN sequence encoded by 5′-CACGGTAAT-3′	This study
pET-ng3(c5)-NEXT-taCA	Expression plasmid carrying the NEXT-taCA gene fused with the HGN sequence encoded by 5′-CACGGTAAC-3′	This study
pET-ng3(c6)-NEXT-taCA	Expression plasmid carrying the NEXT-taCA gene fused with the HGN sequence encoded by 5′-CACGGAAAT-3′	This study
pET-ng3(c7)-NEXT-taCA	Expression plasmid carrying the NEXT-taCA gene fused with the HGN sequence encoded by 5′-CACGGAAAC-3′	This study
pET-ng3(c8)-NEXT-taCA	Expression plasmid carrying the NEXT-taCA gene fused with the HGN sequence encoded by 5′-CATGGCAAT-3′	This study
pET-ng3(c9)-NEXT-taCA	Expression plasmid carrying the NEXT-taCA gene fused with the HGN sequence encoded by 5′-CATGGCAAC-3′	This study
pET-ng3(c10)-NEXT-taCA	Expression plasmid carrying the NEXT-taCA gene fused with the HGN sequence encoded by 5′-CATGGGAAT-3′	This study
pET-ng3(c11)-NEXT-taCA	Expression plasmid carrying the NEXT-taCA gene fused with the HGN sequence encoded by 5′-CATGGGAAC-3′	This study
pET-ng3(c12)-NEXT-taCA	Expression plasmid carrying the NEXT-taCA gene fused with the HGN sequence encoded by 5′-CATGGTAAT-3′	This study
pET-ng3(c13)-NEXT-taCA	Expression plasmid carrying the NEXT-taCA gene fused with the HGN sequence encoded by 5′-CATGGTAAC-3′	This study
pET-ng3(c14)-NEXT-taCA	Expression plasmid carrying the NEXT-taCA gene fused with the HGN sequence encoded by 5′-CATGGAAAT-3′	This study
pET-ng3(c15)-NEXT-taCA	Expression plasmid carrying the NEXT-taCA gene fused with the HGN sequence encoded by 5′-CATGGAAAC-3′	This study
pET-ng3-NEXT-GFP	Expression plasmid carrying the NEXT-GFP gene fused with the HGN sequence	This study
**Primers ^1^**		
TEE1-NEXT-Forward	CATATG**AATCACAAAGTG**GCTGTTCAACATAGCAATGC	This study
TEE2-NEXT-Forward	CATATG**AATCACAAAGTG**AGCAATGCCCCATTGAT	This study
SKIK1-NEXT-Forward	CATATG**TCTAAAATAAAA**GCTGTTCAACATAGCAATGC	This study
SKIK2-NEXT-Forward	CATATG**TCTAAAATAAAA**AGCAATGCCCCATTGATTG	This study
ng1-NEXT-Forward	CATATG**CAC**GCTGTTCAACATAGCAATGC	This study
ng2-NEXT-Forward	CATATG**CACGGC**GCTGTTCAACATAGCAATGC	This study
ng3-NEXT-Forward	CATATG**CACGGCAAT**GCTGTTCAACATAGCAATGC	This study
ng5-NEXT-Forward	CATATG**CACGGCAATCACACC**GCTGTTCAACATAGCAATGC	This study
ng7-NEXT-Forward	CATATG**CACGGCAATCACACCCATTGG**GCTGTTCAACATAGCAATGC	This study
ng9-NEXT-Forward	CATATG**CACGGCAATCACACCCATTGGGGCTAT**GCTGTTCAACATAGCAATGC	This study
ng3(c1)-NEXT-Forward	CATATG**CACGGCAAC**GCTGTTCAACATAGCAATGC	This study
ng3(c2)-NEXT-Forward	CATATG**CACGGGAAT**GCTGTTCAACATAGCAATGC	This study
ng3(c3)-NEXT-Forward	CATATG**CACGGGAAC**GCTGTTCAACATAGCAATGC	This study
ng3(c4)-NEXT-Forward	CATATG**CACGGTAAT**GCTGTTCAACATAGCAATGC	This study
ng3(c5)-NEXT-Forward	CATATG**CACGGTAAC**GCTGTTCAACATAGCAATGC	This study
ng3(c6)-NEXT-Forward	CATATG**CACGGAAAT**GCTGTTCAACATAGCAATGC	This study
ng3(c7)-NEXT-Forward	CATATG**CACGGAAAC**GCTGTTCAACATAGCAATGC	This study
ng3(c8)-NEXT-Forward	CATATG**CATGGCAAT**GCTGTTCAACATAGCAATGC	This study
ng3(c9)-NEXT-Forward	CATATG**CATGGCAAC**GCTGTTCAACATAGCAATGC	This study
ng3(c10)-NEXT-Forward	CATATG**CATGGGAAT**GCTGTTCAACATAGCAATGC	This study
ng3(c11)-NEXT-Forward	CATATG**CATGGGAAC**GCTGTTCAACATAGCAATGC	This study
ng3(c12)-NEXT-Forward	CATATG**CATGGTAAT**GCTGTTCAACATAGCAATGC	This study
ng3(c13)-NEXT-Forward	CATATG**CATGGTAAC**GCTGTTCAACATAGCAATGC	This study
ng3(c14)-NEXT-Forward	CATATG**CATGGAAAT**GCTGTTCAACATAGCAATGC	This study
ng3(c15)-NEXT-Forward	CATATG**CATGGAAAC**GCTGTTCAACATAGCAATGC	This study
NEXT-Reverse	CCATGGAGCCTCCACCGCCGCTGCCACCTCCGCCCACAACGGGTTTTGGTTTAG	[14]

^1^ Restriction sites are underlined, and the regions that contain fusion peptides are in bold type.

**Table 2:** DNA and protein sequences of NEXT-taCA used in this study.

Type	Sequence ^1^
DNA	**ATGGCTGTTCAACATAGCAATGCCCCATTGATTGACTTGGGCGCGGAAATGAAAAAACAGCACAAGGAGGCAGCTCCCGAAGGCGCTGCGCCGGCTCAAGGTAAGGCACCTGCCGCGGAAGCCAAAAAAGAAGAAGCACCTAAACCAAAACCCGTTGTG***GGCGGAGGTGGCAGCGGCGGTGGAGGCTCC*ATGGGTGGTGGCGCTCATTGGGGTTACTCTGGCTCCATTGGCCCGGAACATTGGGGTGACCTGTCGCCGGAATACCTGATGTGCAAAATCGGTAAAAACCAGTCACCGATTGATATCAATTCGGCGGACGCCGTGAAAGCATGCCTGGCTCCTGTTAGTGTCTATTACGTTTCCGATGCAAAATATGTGGTTAACAATGGCCATACCATTAAGGTCGTGATGGGCGGTCGTGGCTATGTTGTCGTGGACGGTAAACGCTTTTACCTGAAGCAATTTCATTTCCACGCGCCGTCCGAACATACCGTCAACGGTAAACACTACCCGTTTGAAGCCCATTTCGTGCACCTGGATAAGAACGGCAATATCACGGTGCTGGGTGTGTTTTTCAAAGTTGGCAAGGAAAATCCGGAACTGGAAAAAGTCTGGCGTGTGATGCCGGAAGAACCGGGTCAGAAACGTCACCTGACCGCACGTATTGATCCGGAAAAGCTGCTGCCGGAAAACCGTGACTATTACCGCTATAGCGGTTCTCTGACCACGCCGCCGTGTAGCGAAGGCGTTCGCTGGATCGTCTTTAAAGAACCGGTGGAAATGTCTCGTGAACAACTGGAAAAATTCCGCAAGGTTATGGGTTTTGACAACAATCGTCCGGTCCAGCCGCTGAATGCCCGTAAAGTGATGAAGCTCGAGCACCACCACCACCACCACTGA
Protein	**MAVQHSNAPLIDLGAEMKKQHKEAAPEGAAPAQGKAPAAEAKKEEAPKPKPVV***GGGGSGGGGS*MGGGAHWGYSGSIGPEHWGDLSPEYLMCKIGKNQSPIDINSADAVKACLAPVSVYYVSDAKYVVNNGHTIKVVMGGRGYVVVDGKRFYLKQFHFHAPSEHTVNGKHYPFEAHFVHLDKNGNITVLGVFFKVGKENPELEKVWRVMPEEPGQKRHLTARIDPEKLLPENRDYYRYSGSLTTPPCSEGVRWIVFKEPVEMSREQLEKFRKVMGFDNNRPVQPLNARKVMKLEHHHHHH

^1^ The sequence of the NEXT tag is in bold type and the (GGGGS)_2_ linker sequence is italicized. The His_6_-tag sequence is underlined along with the *Xho*I restriction site.

**Table 3:** Predicted expression level by the UTR designer.

Enzyme Variants	NEXT-taCA	ng3-NEXT-taCA ^2^															
		c0	c1	c2	c3	c4	c5	c6	c7	c8	c9	c10	c11	c12	c13	c14	c15
**Predicted Expression Level ^1^**	997,966	70,038	153,249	233,117	326,072	166,661	364,665	539,417	443,516	70,038	299,832	137,031	539,417	122,529	637,962	208,446	674,660

^1^ The prediction was done by using the UTR designer and the values are presented in an arbitrary unit. ^2^ The c0 corresponds to the variant with the original codons while the variants c1 to c15 correspond to those with the different codon combinations.

## Data Availability

The data presented in this study are available on request from the corresponding author.

## Sample Availability

Samples of the materials are available from the authors.
